# Supplementary material for: Distinct cellular and molecular mechanisms for β3 adrenergic receptor-induced beige adipocyte formation
Source: eLife. 2017 Oct 11;6:e30329. doi: 10.7554/eLife.30329 (PMC5667933; doi:10.7554/eLife.30329)
Supplement: Supplementary file 1. [file elife-30329-supp1.docx]

Supplementary file 1

| **Gene** | **Forward** | **Reverse** |
| --- | --- | --- |
| *ACTA2/SMA* | GTCCCAGACATCAGGGAGTAA | TCGGATACTTCAGCGTCAGGA |
| *ADRB1* | CTCGTCCGTCGTCTCCTTCTAC | GTCGATCTTCTTTACCTGTTTTTGG |
| *ADRB2* | TTGCAGTGGATCGCTATGTTG | TGACCACTCGGGCCTTATTCT |
| *ADRB3* | CCTTCAACCCGGTCATCTAC | GAAGATGGGGATCAAGCAAGC |
| *CD24* | ACGGAGCGGACATGGGCAGA | TTGGTTGCAGTAAATCTGCGTGGGT |
| *CD45* | ATGGTCCTCTGAATAAAGCCCA | TCAGCACTATTGGTAGGCTCC |
| *Cidea* | TCTGCAATCCCATGAATGTC | CAGTGATTTAAGAGACGCGG |
| *Cox8b* | TGTGGGGATCTCAGCCATAGT | AGTGGGCTAAGACCCATCCTG |
| *Dio2* | ACACTGGAATTGGGAGCATC | ATGCTGACCTCAGAAGGGCT |
| *Elovl3* | TTCTCACGCGGGTTAAAAATGG | GAGCAACAGATAGACGACCAC |
| *Myh11* | AAGCTGCGGCTAGAGGTCA | CCCTCCCTTTGATGGCTGAG |
| *NG2 (CSPG4)* | GGGCTGTGCTGTCTGTTGA | TGATTCCCTTCAGGTAAGGCA |
| *PDGFRα* | TCCATGCTAGACTCAGAAGTCA | TCCCGGTGGACACAATTTTTC |
| *PDGFRβ* | AGGGGGCGTGATGACTAGG | TTCCAGGAGTGATACCAGCTT |
| *PGC1α* | TATGGAGTGACATAGAGTGTGCT | CCACTTCAATCCACCCAGAAAG |
| *PPARγ* | TCGCTGATGCACTGCCTATG | GAGAGGTCCACAGAGCTGATT |
| *PRDM16* | ACACGCCAGTTCTCCAACCTGT | TGCTTGTTGAGGGAGGAGGTA |
| *Pref-1 (DLK1)* | CCCAGGTGAGCTTCGAGTG | GGAGAGGGGTACTCTTGTTGAG |
| *Rn18S* | GTAACCCGTTGAACCCCATT | CCATCCAATCGGTAGTAGCG |
| *TBX1* | CTGTGGGACGAGTTCAATCAG | TTGTCATCTACGGGCACAAAG |
| *UCP1* | CGACTCAGTCCAAGAGTACTTCTCTT | GCCGGCTGAGATCTTGTTTC |
| *ZFP423* | CAAGAGGAGAGAAATGAGGACGA | AGTGATCGCAGGTGTAAATTGAC |
